# Acute Liver Injury Induced by Sitagliptin: Report of Two Cases and Review of Literature

**DOI:** 10.7759/cureus.2776

**Published:** 2018-06-11

**Authors:** Amir Shahbaz, Kashif Aziz, Muhammad Umair, Mohaddeseh Sharifzadeh, Issac Sachmechi

**Affiliations:** 1 Internal Medicine, Icahn School of Medicine at Mount Sinai Queen Hospital Center, New York, USA

**Keywords:** sitagliptin, drug-induced liver injury, side effects, type 2 diabetes mellitus

## Abstract

We present two cases of acute liver injury associated with sitagliptin. The first case was a 58-year-old male with a history of poorly controlled type 2 diabetes mellitus and hyperlipidemia. Sitagliptin was added for better control of diabetes. After initiation of sitagliptin, the patient's serum alanine aminotransferase (ALT) and aspartate aminotransferase (AST) levels increased gradually over a period of six months. The second case was a 44-year-old female with type 2 diabetes mellitus, and she experienced a more than ten-fold elevation in ALT and AST levels after starting sitagliptin therapy. Both patients did not have any history of alcohol abuse, acetaminophen use, or chronic liver disease. In the literature review, the reported magnitude of liver enzyme derangement with sitagliptin is generally mild and transient (two-fold upper limit of normal). We believed that the acute derangement of ALT and AST in our patients was due to sitagliptin since the ALT and AST normalized shortly after sitagliptin was discontinued and remained at baseline after resuming all other medications. Further research is needed to understand the mechanism of dipeptidyl peptidase 4 (DPP-4) inhibitors associated liver injury.

## Introduction

Diabetes mellitus is either type 1, caused by inherited and/or acquired deficiency in the production of insulin, or type 2, caused by a resistance to the effectiveness of insulin. Sitagliptin is a dipeptidyl-peptidase-4 (DPP-4) inhibitor, an oral antihyperglycemic agent. It blocks the degradation of incretin and enhances incretin levels, which stimulate insulin secretion and decreases glucagon production. Sitagliptin is effective in lowering glycosylated hemoglobin (Hb A1C) and improves fasting and postprandial glucose levels [1]. DPP-4 inhibitors have a favorable safety profile in clinical trials. Further investigation is needed as rare side effects arise in post-marketing surveillance. Some important but rare side effects of DPP-4 inhibitors are a potential risk for pancreatitis and thyroid cancer [2]. In clinical practice, a drug-induced liver injury is not a common side effect in patients taking DPP-4 inhibitors. We reported two cases of drug-induced hepatic injury caused by sitagliptin.

## Case presentation

The first case was a 58-year-old male with poorly controlled type 2 diabetes mellitus and hyperlipidemia who experienced a six-fold increase in his serum alanine aminotransferase (ALT) and aspartate aminotransferase (AST) levels from baseline. The patient had been taking pravastatin and ezetimibe for a few years for hyperlipidemia. Sitagliptin was recently added to the patient's medical regimen for better control of his diabetes. After initiating sitagliptin, the patient's ALT and AST increased gradually over a period of six months. Work up was done to find the possible cause of the abnormal liver function tests (LFTs). Hepatitis serologies were negative. An abdominal sonogram was also negative for gallstones. In the absence of any pathological cause, a diagnosis of drug-induced liver injury was made. All medications were discontinued and LFTs were done on regular follow-up visits. After discontinuing sitagliptin, pravastatin, and ezetimibe, the patient's ALT and AST returned to baseline levels. Resumption of pravastatin and ezetimibe was not associated with the elevation in ALT and AST levels. When we rechallenged the patient with sitagliptin, we observed a  five-fold increase in the levels of serum ALT and AST from the baseline that became normal after discontinuation of sitagliptin. The second case was a 44-year-old female with type 2 diabetes who experienced a more than ten-fold elevation in ALT and AST levels after six months of sitagliptin therapy. Further workup revealed a negative hepatitis B-surface antigen with a normal liver sonogram. The patient's ALT and AST levels returned to normal after discontinuing sitagliptin, pioglitazone, and rosuvastatin. Resumption of pioglitazone and rosuvastatin was not associated with elevation in ALT and AST levels. The patient refused the rechallenge with sitagliptin.

## Discussion

The efficacy and safety of sitagliptin have been well documented in clinical trials of patients with type 2 diabetes, either as a monotherapy or in combination with other oral hypoglycemic agents. A review of the literature identified only two case reports of elevated hepatic transaminases associated with sitagliptin [3-4].  However, clinical trials of sitagliptin monotherapy or in combination with other oral hypoglycemic agents have demonstrated no change or a slight decrease in AST or ALT levels from the baseline with sitagliptin treatment [5].

Raz et al. studied the safety and efficacy of 100 mg and 200 mg doses of sitagliptin as monotherapy versus placebo and found no meaningful change from the baseline in AST or ALT levels between the treatment groups [5]. Nauck et al. conducted a study to compare the effect of sitagliptin 100 mg and metformin, or glipizide and metformin. The researchers found a slight mean decrease in ALT in the sitagliptin group versus a slight increase in ALT in the glipizide group, which was not statistically significant [6]. 

 We present two cases of acute liver injury caused by sitagliptin. Other causes of elevated liver enzymes, such as acute viral hepatitis, nonalcoholic steatohepatitis, autoimmune hepatitis, or the consumption of alcohol and hepatotoxic drugs were ruled out. Also important were the observed disappearance of symptoms and the return of elevated liver enzymes to baseline after discontinuation of the sitagliptin. DPP-4 is expressed in the liver and, in fact, DPP-4 has been implicated in the pathogenesis of several chronic liver diseases [7]. For these reasons, DPP-4 inhibition has been proposed as beneficial in chronic liver diseases [8]. Further research is needed to explore the possible underlying mechanism of DPP-4 inhibitors associated liver injury. While reviewing the literature, we also found one case report of potential linagliptin and vildagliptin induced liver toxicity [9-10]. This suggests that liver toxicity might be a class effect of DPP-4 inhibitors. Discontinuation of the offending agent will bring about a dramatic improvement in symptoms and prevent adverse outcomes [11].

## Conclusions

Drugs are an important cause of acute liver injury. Physicians should take a detailed and thorough history of drug intake in any patient presenting with abnormal liver function tests. When a diabetic patient using sitagliptin presents with unexplained elevated liver enzymes, then drug-induced hepatic injury should be suspected. We recommend that all patients receiving sitagliptin should have their liver function assessed periodically. Sitagliptin administration should be stopped immediately at the onset of abnormal liver function if no other obvious cause for the abnormal liver function is present.
